# A Hybrid Route Selection Scheme for 5G Network Scenarios: An Experimental Approach

**DOI:** 10.3390/s22166021

**Published:** 2022-08-12

**Authors:** Mohammad Kazem Chamran, Kok-Lim Alvin Yau, Mee Hong Ling, Yung-Wey Chong

**Affiliations:** 1 Department of Computing and Information Systems, School of Engineering and Technology, Sunway University, Petaling Jaya 47500, Malaysia; 2 Lee Kong Chian Faculty of Engineering & Science, Universiti Tunku Abdul Rahman (UTAR), Kajang 43200, Malaysia; 3 National Advanced IPv6 Centre, Universiti Sains Malaysia (USM), Penang 11800, Malaysia

**Keywords:** hybrid route selection, 5G, reinforcement learning, USRP/GNU radio, Raspberry Pi, centralized route selection, distributed route selection, cognitive radio, D2D communication, femtocell node and base station

## Abstract

With the significant rise in demand for network utilization, such as data transmission and device-to-device (D2D) communication, fifth-generation (5G) networks have been proposed to fill the demand. Deploying 5G enhances the utilization of network channels and allows users to exploit licensed channels in the absence of primary users (PUs). In this paper, a hybrid route selection mechanism is proposed, and it allows the central controller (CC) to evaluate the route map proactively in a centralized manner for source nodes. In contrast, source nodes are enabled to make their own decisions reactively and select a route in a distributed manner. D2D communication is preferred, which helps networks to offload traffic from the control plane to the data plane. In addition to the theoretical analysis, a real testbed was set up for the proof of concept; it was composed of eleven nodes with independent processing units. Experiment results showed improvements in traffic offloading, higher utilization of network channels, and a lower interference level between primary and secondary users. Packet delivery ratio and end-to-end delay were affected due to a higher number of intermediate nodes and the dynamicity of PU activities.

## 1. Introduction

Fifth-generation (5G) has been envisaged as the next-generation cellular network for deploying, supporting, and scaling new technologies, including augmented reality, driver-less vehicles, Internet of Things, smart cities, virtual reality, and 3D video streaming services. Nevertheless, *three* main characteristics of the next-generation network scenario have posed main challenges to the realization of 5G. Firstly, *dynamicity of channel availability*, in which the operating channels, particularly the licensed channels, can be randomly occupied by licensed users (or primary users, PUs); consequently, unlicensed users (or secondary users, SUs) must search for and use the licensed channels in an opportunistic manner [1,2,3,4,5]. Secondly, *heterogeneity,* in which the network consists of different types of network cells (e.g., *macrocells* and *small cells*) and different types of nodes (e.g., using different operating channels and transmission power levels); consequently, nodes must adapt to a diversified operating environment [6,7,8,9,10]. Thirdly, *ultra-densification,* in which there are a large number of base stations (BSs), particularly small cells, and nodes in an area; consequently, nodes must search within nodes and BSs to find a route with the least traffic intensity in order to maximize traffic offloading [7,11,12,13].

**Figure 1:**
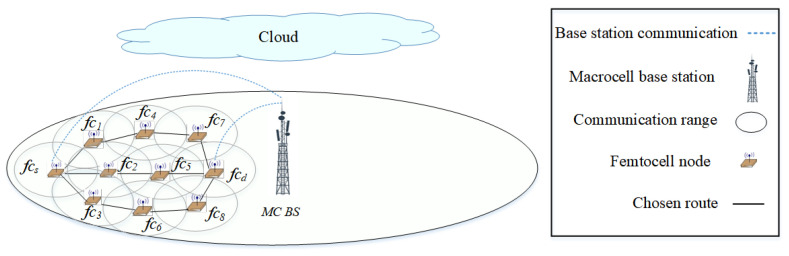
A multi-tiered 5G network. The source node $fc_{s}$ establishes a route to the destination node $fc_{d}$. The optional routes are also shown.

**Figure 2:**
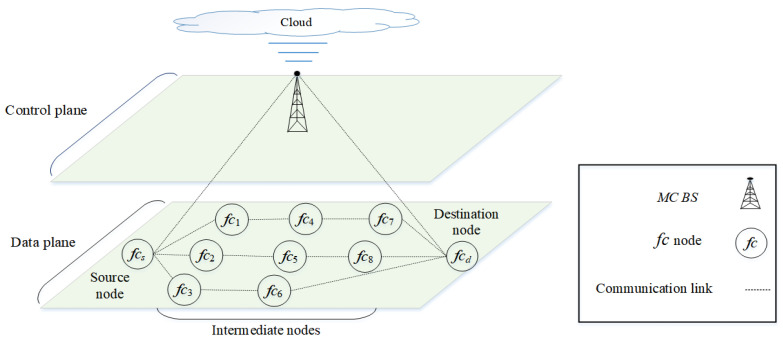
Control and data plane separation in a multi-tiered 5G network. An equivalent network is shown in Figure 1.

The fifth generation (5G) is a multi-tier cellular network, as shown in Figure 1. There are different types of network cells, such as *macrocells* and *femtocells*. Macrocells have the broadest coverage, followed by femtocells (e.g., 30 m radius). In Figure 1, the BSs of femtocells $fc_{1},fc_{2},\ldots ,fc_{8}$ are scattered within the transmission range of a macrocell base station (MC BS). The BSs of the macrocell and femtocells can communicate with each other either directly or via multiple hops. Small cells deployed outside the transmission range of macrocells can help to improve network coverage. Due to the different characteristics of the network cells, the network can be segregated into two main layers, as shown in Figure 2. The *macrocell layer* uses lower frequency bands with a higher transmission power to provide a longer transmission range; however, the channel capacity is lower at lower frequency bands. The small-cell layer (i.e., the *femtocell layer*) uses higher frequency bands (e.g., millimeter wave (mm-wave) or above 30 GHz [14]) with a lower transmission power to provide a higher channel capacity; however, the transmission range is shorter at higher frequency bands. In terms of the dynamicity of channel availability, the macrocell layer may use channels with a higher number of white spaces, given a limited number of available licensed channels. In terms of heterogeneity and ultra-densification, these characteristics are more prevalent in small cells as a large number of small cells must be deployed to provide connections to heterogeneous nodes (or user equipment). Some of the main characteristics of 5G architecture are *control plane* and *data plane* separation as shown in Figure 2. The *control plane*, which includes the macrocell layer, contains a central controller (CC) that (a) collects network-wide information (e.g., network topology comprised of nodes, links, and channel availability) from all nodes in the network and maintains the information (e.g., in a routing table); (b) performs global-level tasks (e.g., determines routes); and c) disseminates information (e.g., prioritizes routes) to nodes in the network (i.e., source node $fc_{s}$). The *data plane*, which includes the small-cell layer, performs local-level tasks (e.g., selects a route based on a set of available and prioritized routes, and the availability of *device-to-device (D2D)* communication) [15,16].

There are two main features of 5G. Firstly, *dynamic channel access*, whereby a SU node senses underutilized channels in licensed channels (or white spaces) owned by PUs, and subsequently accesses the white spaces in an opportunistic manner without causing unacceptable interference to PU activities in order to improve the overall spectrum utilization. Secondly, D2D communication, whereby a node can communicate directly with its neighboring nodes without passing through a BS, which helps to (a) offload traffic from highly-utilized BSs, particularly the MC BSs, to improve load balancing and reduce traffic congestion at BSs, and (b) extend coverage.

This paper proposes a cognition-inspired hybrid route selection scheme called CenTri for 5G network scenarios to embrace these two main features. CenTri consists of centralized and distributed route selection mechanisms and establishes multi-hop routes across the macrocell and small-cell layers. The centralized route selection mechanism by the CC adopts dynamic channel access to address the dynamicity of channel availability. In contrast, the distributed route selection mechanism adopts D2D to address heterogeneity and ultra-densification. Cognition enables a decision-maker (or an agent) to observe the respective operating environment and learn about the right decisions on the route selection in the operating environment at any time instant. In CenTri, cognition is embedded in the source node, which is the final ‘decision maker’ to learn about and select the best possible route with a high amount of white spaces to facilitate the traffic offload from the macrocell through traffic distribution to the femtocell layer. The CC sends a list of routes, which are ranked (or prioritized) based on the route length (or the number of hops), to the source node. The source node reranks the routes while considering the traffic offload, using the femtocell layer and the congestion level of the MC BS.

### 1.1. Why Is a Hybrid Route Selection Scheme Crucial to 5G Networks?

While separate centralized mechanisms [6,7,17] and distributed mechanisms [18,19,20] for route selection are shown to improve network performance, the benefits of combining both mechanisms are yet to be discovered in the context of 5G, and so a hybrid route selection scheme is the focus of this paper. Intuitively, a hybrid route selection scheme can address the intrinsic limitations of each mechanism. In the centralized mechanism, the computational complexity and routing overhead increase with the number of nodes in the network (or node density) as each source node in the *data plane* must receive information from the CC and then discover and maintain routes (e.g., update the routing table) [9,21]. In the distributed mechanism, the routing overhead increases as each node in the *data plane* must report the information availability (i.e., the available channels and the bottleneck link rate) with neighboring source nodes [12,22,23]. The issues observed in both centralized and distributed mechanisms intensify with the increased dynamicity of channel availability, network heterogeneity, and ultra-densification. Our proposed hybrid mechanism uses both centralized and distributed mechanisms. The distributed mechanism performs the traditional centralized tasks, particularly those using highly dynamic information in a distributed manner to minimize high computational complexities at the CC. Naturally, the routing overhead reduces because BSs and nodes do not send dynamic information (e.g., the traffic level) to the CC. The centralized mechanism performs the traditional distributed tasks, particularly those requiring frequent information exchange among BSs and nodes in a centralized manner to minimize high routing overheads at BSs and nodes. Naturally, the routing overhead reduces because BSs and nodes do not exchange dynamic information among themselves.

CenTri consists of *centralized* and *distributed* mechanisms for route selection. The centralized mechanism is embedded in a CC in the control plane, gathers and maintains network-wide and less dynamic information (i.e., available routes), and selects routes across different layers (i.e., the macrocell and small-cell layers). Subsequently, the source node makes routing decisions based on the list of routes provided by the CC. The routing decisions prioritize routes with fewer PUs in the data plane and D2D communication. The purpose is to minimize SU interference to PUs at the global level. In addition, being more stable, the routes can be established in a proactive manner to serve as backbone routes for different source–destination pairs, contributing to a reduced routing overhead required in the route discovery and maintenance. The distributed mechanism, which is embedded in each BS and node in the data plane, gathers and maintains neighborhood (or local) and more dynamic information (i.e., available routes and traffic levels of nodes in range), and selects intra- or inter-cell routes in the small-cell layer. The purpose is to maximize traffic offload from BSs, particularly the MC BSs, at the local level. Being more dynamic, the routes can be established via D2D among BSs and nodes in the small-cell layer in a reactive (or on-demand) manner to offload traffic from the macrocell layer.

### 1.2. Why Is Cognition Crucial to the Hybrid Route Selection Scheme in 5G Networks?

Due to the need for interaction with the operating environment, the majority of testbeds using USRP have adopted Q-learning to adapt to the environment. Q-learning is the preferred approach because it does not need datasets required in supervised machine learning approaches and does not explore the underlying pattern or relevancy. A clear justification is needed (e.g., utilized or unutilized), which is why an unsupervised ML is not preferred [24,25,26]. For more details, refer to Section 3.1.

Due to the dynamicity of the operating environment, it is necessary to practice constant learning to achieve optimal network performance as time passes. As different types of network cells have different characteristics, the dynamicity level varies across the macrocell and small-cell layers. For example, the dynamicity of channel availability is lower in the macrocell layer as channels with a higher number of white spaces have been assigned to the layer. Therefore, a single set of rules or policies would less likely be optimal when applied across the entire network. In CenTri, BSs and nodes embrace a popular cognition approach called reinforcement learning (RL).

### 1.3. CenTri as a Hybrid Route Selection Scheme: An Overview

In CenTri, the centralized mechanism enables a CC to establish backbone routes that minimize SU interference to PUs based on network-wide information (i.e., channel availability of routes). The backbone routes can be used by different source–destination pairs. Nevertheless, the centralized mechanism has two shortcomings: (a) the CC is not sensitive to the dynamicity of the local environment (i.e., the traffic level of each source node towards its destination node) and (b) the routes predetermined and disseminated to source nodes by the CC may suggest the best route being the shortest one, which goes through the backbone route, causing an increased traffic load at the MC BS. The distributed mechanism enables nodes of small cells to revise routing decisions received from the CC to maximize traffic offload from MC BSs based on local information (i.e., available routes and traffic levels of D2D routes).

In the small-cell layer, a source node selects a route based on four priority levels, which helps to offload traffic from the macrocell layer: the *first* (or highest) priority is communication via the femtocell layer (through a D2D route), the *second* priority is communication via the route with minimum interference from PUs, the *third* priority is communication via the route with the minimum number of hops, and the *fourth* (or last) priority is communication via the backbone route. A single multi-hop route can consist of links belonging to different layers. For example, the communication through the backbone route connects a source node of the femtocell layer to a BS of the macrocell layer. It then transmits the data to the destination node in the femtocell layer. In Figure 1, the $fc_{s}-fc_{2}-fc_{5}-fc_{d}$ route is preferred compared to $fc_{s}-mc-fc_{d}$ used in the traditional network.

RL is embedded in the source nodes of the femtocell layer so that the right decisions can be made on the route selection at the local level to reduce the global workload of the CC.

### 1.4. Reinforcement Learning: An Overview

Q-learning, which is a popular RL approach, enables an agent (or decision maker) to gain knowledge independently in order to take the right action at the right time in its operating environment for individual performance enhancement. At any time instant *t*, an agent *i* observes its operating environment in the form of state $s_{t}^{i}$, and then selects and takes action $a_{t}^{i}$ in the operating environment. At the next time instant t+1, the agent *i* receives a reward $r_{t+1}^{i}\left(s_{t+1}^{i}\right)$ and the state changes to $s_{t+1}^{i}\leftarrow s_{t}^{i}$. The Q-value $Q_{t}^{i}\left(s_{t}^{i},a_{t}^{i}\right)$, which represents the long-term reward of a state–action pair $\left(s_{t}^{i},a_{t}^{i}\right)$ of an agent *i*, is updated using the Q-function, as follows:

(1) $$\begin{array}{cc} Q_{t+1}^{i}\left(s_{t}^{i},a_{t}^{i}\right) & =Q_{t}^{i}\left(s_{t}^{i},a_{t}^{i}\right)+\alpha [r_{t+1}^{i}\left(s_{t+1}^{i}\right)\\ \; & \;\;\;+\gamma (Q_{t+1}^{i}\left(s_{t}^{i},a_{t}^{i}\right)-Q_{t}^{i}\left(s_{t}^{i},a_{t}^{i}\right))] \end{array}$$

where 0≤α≤1 represents the learning rate and 0≤γ≤1 represents the discount factor, which is the next state–action pair emphasizing the future reward. The discount reward always has a lesser weight than the immediate reward.

### 1.5. Contributions

Common routing mechanisms, such as route requests and route replies, have been well investigated in the literature [27,28,29,30]. This paper focuses on route selection and makes three contributions. *Firstly*, a hybrid route selection scheme called CenTri is proposed to select routes that minimize SU interference to PUs in available licensed channels in 5G networks characterized by the dynamicity of channel availability and ultra-densification. The purposes are to: a) improve load balancing through traffic offload from the macrocell layer to the small-cell layer; and b) the overall spectrum utilization. *Secondly*, RL models and algorithms are proposed for CenTri. *Thirdly*, the issues associated with implementing CenTri on a real-world platform consisting of heterogeneous nodes embedded with universal software radio peripheral (USRP) units are presented.

### 1.6. Organization of This Paper

The rest of this paper is organized as follows. Section 2 presents the related work. Section 3 presents the system model. Section 4 presents the CenTri RL model and algorithm. Section 5 presents the results and discussion.

## 2. Related Work

This section presents related work on routing in 5G networks, hybrid route selection schemes, the application of RL to hybrid route selection schemes, and the implementation of schemes on a real-world 5G platform consisting of USRP units.

### 2.1. Routing in 5G

Based on the functions of the route selection schemes in 5G networks, there are two main categories: (a) traffic offloading [9,21,23,31,32]; and (b) traffic splitting by selecting routes comprised of nodes in the femtocell layer [21,31,33].

#### 2.1.1. Traffic Offloading

Traffic offloading allows traffic to be offloaded from highly utilized BSs, particularly MC BSs to other BSs (e.g., in femtocells) in order to improve load balancing and reduce traffic congestion at BSs of the upper-tier (i.e., the macrocell layer). This can be achieved via D2D communication.

In [34], a distributed channel allocation scheme is proposed to offload traffic to D2D nodes in small cells to avoid (or reduce) interference between licensed and unlicensed users. The scheme addresses network heterogeneity (i.e., small-cell BSs and D2D users) and potential interference from unlicensed users. For channel allocation, channels are allocated to licensed users, and then the available channels are shared among D2D users communicating within the range of a small-cell network called a coalition. Therefore, the priority of spectrum sharing is higher among D2D devices in a coalition. The proposed scheme increases the throughput and network spectrum utilization due to better channel allocation and route selection.

In [9], a distributed route selection scheme is proposed to offload traffic from highly congested BSs to less congested BSs via D2D communication in order to achieve load balancing. The scheme addresses network heterogeneity (i.e., macrocell and small-cell BSs) and ultra-densification. Traffic can be offloaded from: (a) congested MS BSs to less congested small cells; (b) congested small-cell BSs to less congested small-cell BSs; and (c) from congested small-cell BSs to less congested MC BSs. Deploying a large number of small cells can lead to ultra-densification. Dijkstra’s algorithm [35] is applied to determine the shortest route (which has the least number of nodes and hops) towards a destination node. The proposed scheme increases the throughput due to better traffic distribution among the network cells.

In [32], a distributed route selection scheme is proposed to offload traffic from nodes with lesser resources (i.e., residual energy and memory) to those with more resources via D2D communication in order to improve network performance. The scheme addresses the dynamicity of channel availability and network heterogeneity, whereby nodes have different amounts of resources. Since routes have different resource requirements, nodes with higher residual energy and memory are selected to establish resource-intensive routes to deliver more traffic; however, once their resources drop below a certain threshold, the nodes are selected for less resource-intensive routes. The proposed scheme is shown to increase the packet delivery rate as nodes with more resources are selected for packet forwarding.

In [23], a distributed route selection scheme is proposed to offload traffic from nodes with low residual energy to those with higher residual energy yet less traffic load via D2D communication in order to prolong the network lifetime. The scheme addresses the dynamicity of channel availability and network heterogeneity, whereby nodes have different residual energy levels. In other words, nodes with higher residual energy levels are well utilized, while nodes with lower residual energy levels serve the backup role. Rerouting is performed from time to time as the residual energy levels at different nodes reduce at different rates, and the traffic load is dynamic in nature. Among the available routes, the one with higher residual energy and lower end-to-end delay, in terms of the number of hops, is selected to deliver traffic. The proposed scheme is shown to increase the network lifetime and packet delivery rate as load balancing is achieved among nodes with different residual energy levels throughout the network.

#### 2.1.2. Traffic Splitting among Available Routes

Traffic splitting allows traffic to be split and distributed among different routes in order to improve load balancing and reduce traffic congestion at BSs serving large traffic streams. Traffic splitting is possible in 5G networks as the CC is aware of source nodes containing a particular traffic stream, as well as the available routes towards a destination.

In [21,31], a centralized route selection scheme is proposed to split and distribute large traffic streams among available routes in order to achieve load balancing. The scheme addresses the dynamicity of channel availability. The CC: (a) gathers network-wide information (i.e., network topology comprised of nodes and links, resources comprised of channel availability, and traffic patterns); (b) identifies source nodes that possess a particular traffic stream requested by a destination node; (c) estimates the channel capacity and end-to-end delay of routes established between a pair of source and destination nodes; and (d) dynamically segregates a traffic stream into different fractions and sizes contributed by different source nodes based on the channel capacity and the amount of traffic of the candidate routes. Based on the routing decisions made by the CC, a source node sends parts of a traffic stream in fractions to a destination node. Intermediate nodes that receive the fractions from different source nodes aggregate them, and subsequently split them among available outgoing links towards a destination based on the shortest route, in terms of the number of links and the current traffic amount of each route. The destination node aggregates the fractions to obtain the original traffic stream. The proposed scheme is shown to reduce end-to-end delay. A similar traffic splitting scheme has been presented in [33], although the intermediate nodes that receive the fractions do not aggregate and split them. In [36], intermediate nodes receive updated routing tables to address the split traffic to the right destination. The split traffic scheme is shown to improve throughput and reduce handover and end-to-end delay.

#### 2.1.3. CenTri for Achieving Traffic Offloading

CenTri enables BSs and nodes of small cells to maximize the traffic offload from MC BSs (see Section 2.1.1). In the next section, the 5G characteristics of CenTri are presented.

### 2.2. Hybrid Routing Schemes in Wireless Networks

Hybrid route selection schemes consist of centralized and distributed mechanisms that cooperate to determine the least cost routes (e.g., with lower traffic loads and the number of hops).

In [37], a hybrid route selection scheme consists of a centralized mechanism at the control plane and a distributed mechanism at the data plane to shift traffic intensity from congested routes to routes with less traffic. The route update procedure consists of two mechanisms: (a) proactive route updates from the CC with minimum delay; and (b) reactive route updates from the CC after data transmission completes in a node in the data plane. Therefore, the proposed algorithm provides an ingress threshold to determine the congestion level of each node before a route update takes place. The proposed scheme is shown to improve load balancing and increase the network throughput.

In [38,39], a hybrid route selection scheme, which consists of proactive and reactive mechanisms, is proposed to establish routes among two types of nodes, namely static and mobile nodes. Firstly, *static nodes*, which have lower dynamicity and energy constraints, perform: (a) proactive route selection to establish backbone routes towards a gateway node for internet access; and (b) reactive route selection to establish routes with mobile nodes. Secondly, *mobile nodes*, which have higher dynamicity and energy constraints, perform reactive route selections to establish better routes (i.e., with lower traffic loads and higher residual energy) towards gateway nodes. The proposed approach is shown to increase throughput, and reduce packet loss rate and end-to-end delay. A similar hybrid route selection scheme was proposed in [39]. The *mobile nodes* perform the reactive route selection to establish routes with a higher number of static nodes (or a lower number of mobile nodes) in order to improve route stability; the proposed scheme is shown to improve throughput and reduce the delay incurred in the route selection and the energy consumption of mobile nodes.

In [40,41], a hybrid route selection scheme, which consists of centralized and distributed mechanisms, is proposed to offload traffic from routes with busier nodes to routes with less occupied nodes. Nodes are segregated into groups, and several nodes in a group are selected as group leaders to communicate with neighboring groups. For intra-group communication, each node selects a route with a lower number of hops and traffic load in a distributed manner. For inter-group communication, with more than a single group leader, a source node can establish more than a single route towards a destination node. The CC selects a route with the lowest number of hops or traffic load and communicates the selected route to the source node. The proposed scheme is shown to increase throughput and channel utilization, as well as reduce the packet loss rate caused by route breakages.

CenTri is a hybrid route selection scheme that consists of centralized and distributed mechanisms. The centralized mechanism adopts a proactive approach to establish backbone routes using network-wide information in order to search for and use white spaces. In contrast, the distributed mechanism adopts a reactive approach to further improve the routes using local information in order to perform traffic offloading. In comparison with existing works, (a) CenTri considers heterogeneous nodes and networks as seen in 5G, it is different from [37,38,39,40,41] in terms of the control plane and data plane separation and the platform experimental setup with up to 11 nodes; (b) the traffic offloading mechanism makes it different from [9,23,32,34] as the platform offers a coexistence of proactive–centralized and reactive distributed; (c) the use of D2D communication of the femtocell with a backbone route and the capability of learning the most efficient route with the least presence of PUs and dynamicity of channel availability, which distinguish the platform from [20,23,37,42,43,44,45,46].

### 2.3. Application of Reinforcement Learning to Hybrid Routing

In [42], a hybrid route selection scheme comprised of proactive and reactive mechanisms is proposed to establish stable routes from a source node to a destination node. There are two zones: (a) the intra-zone consists of nodes located within the transmission range of the node, and (b) the inter-zone consists of nodes located out of the transmission range of the node. The proactive mechanism establishes routes to the inter-zone nodes, while the reactive mechanism establishes routes to the intra-zone nodes. The traditional Q-learning approach is embedded in the proactive mechanism of each node to adjust the routing period, which is the time period of the next route discovery since the last one. A longer routing period is suitable for a network with lower dynamicity since existing routes are likely to function. In comparison, a shorter routing period is suitable for networks with higher dynamicity since existing routes are likely to be broken, so new routes must be established. By adjusting the routing period, the number of route discoveries can be adjusted according to network dynamics (i.e., mobility, residual energy level, and channel availability). In this RL scheme, the state represents the destination node, the action represents the selection of the routing period, and the reward represents the network dynamics. The proposed scheme is shown to increase the packet delivery rate and reduce routing overhead.

In [47], a hybrid route selection scheme, composed of learning- and non-learning-based mechanisms, is proposed to establish stable routes from a source node to a destination node. The non-learning-based mechanism establishes least-cost routes using Dijkstra’s algorithm during normal operation, while the learning-based mechanism estimates the link cost when it changes. Subsequently, the estimated link cost is used by the non-learning-based mechanism to revise its routes. The traditional Q-learning approach is embedded in the learning-based mechanism of each node to provide accurate estimations of the link cost in order to minimize route oscillation, whereby routes oscillate, between less utilized and favorable and highly utilized and unfavorable as time goes by. In this RL scheme, the state represents a link, the action represents the selection of the link cost, and the reward represents factors affecting the link cost. The proposed scheme is shown to reduce the packet loss rate and end-to-end delay.

In CenTri, the CC in the control plane establishes backbone routes in order to minimize SU interference to PUs. The nodes and BSs in the control plane have less dynamicity while the nodes and BSs in the data plane have high dynamicity. Therefore, the RL model is embedded in the distributed mechanism to enable BSs and nodes in the data plane to revise the proactive routes in order to offload traffic from highly utilized nodes and BSs, particularly MC BSs, to less utilized BSs, particularly small-cell BSs. The RL model of the distributed mechanism uses more dynamic local information (i.e., traffic levels at the BSs). In Section 4, the CenTri model and algorithm are presented.

### 2.4. Testbed Implementation of a Hybrid Route Selection Scheme

Universal software radio peripheral (USRP) testbeds have been deployed to perform route selection for 5G and cognitive radio networks. Together with an open-source software toolkit, namely GNU radio [48], signals are generated and various processes, including encoding, decoding, and modulation are performed. In this work, CenTri is implemented on a USRP/GNU radio testbed for 5G networks. While most experimental studies focus on data links and physical layer investigations, including interference mitigation [13,14,49], channel sensing [50], and power allocation  [51,52], this paper focuses on network layer implementation. There are six nodes used in [43,44], eight nodes in [20], and ten nodes in [46].

In [20,43,44,46], the route selection scheme enables a SU to establish stable routes in the presence of PU activities in a multi-hop cognitive radio network. The routing metrics are channel availability [20,44,46,53], channel quality [43,44,46], interference among SUs [43], and the number of hops of the route [20]. In [46], RL is embedded in each node to establish stable routes with the highest possible channel available time. The SU must perform channel sensing from time to time to measure channel availability based on the number of available channels and the number of route switches. The SUs can learn about the availability of white spaces via channel sensing in opportunistic channel access, or via direct communication with PUs in channel leasing. To implement a larger multi-hop network with more nodes (e.g., >10 nodes), the USRP/GNU radio units are connected to a single computer via a switch [46], which helps to reduce hardware and software delays. The proposed schemes have been shown to increase throughput [20,43,44] (or packet delivery rate [46]), as well as reduce the end-to-end delay [20,43,44], the number of channel switches [20,46], the number of route breakages [46], and routing overhead caused by rerouting [43,44].

In this work, a heterogeneous network is considered in which there are up to eleven USRP/GNU radio nodes from different layers, including the macrocell and femtocell layers. Hence, the nodes have different characteristics in terms of the choice of operating channels, transmission power, and transmission range. Each node consists of a USRP/GNU radio unit, which serves as the RF front end, connected to a mini-computer Raspberry Pi3 B+, which is programmed to run CenTri. Thus, the testbed of this work extends those in the previous works [43,44,46], whereby the USRPs/GNU radio units are wire-connected with a single computer via a switch to emulate a common control channel. To the best of our knowledge, this is the first testbed implementation for a hybrid route selection scheme.

## 3. System Model

There is a set of channels $C=\{c_{1},c_{2},\ldots ,c_{\left|C\right|}\}$, each $c_{i}$ is occupied by PU $p_{i}\in P=\left\{p_{1},p_{2},\ldots ,p_{\left|P\right|}\right\}$. A multi-tier 5G network shown in Figure 1 and Figure 2 is considered. The MC BSs $MC=\{mc_{1},mc_{2},\ldots ,mc_{\left|N\right|}\}$ and femtocell nodes and BSs, $FC=\{fc_{1},fc_{2},\ldots ,fc_{\left|N\right|}\}$ can communicate among themselves directly. For instance, a femtocell BS $fc_{1}$ can communicate with a MC BS $mc_{1}$ directly via $fc_{1}-mc_{1}$. A BS can establish a multi-hop route $k_{n}\in K=\left\{k_{1},k_{2},\ldots ,k_{\left|K\right|}\right\}$ across different layers. For instance, a femtocell source node $fc_{s}$ establishes a multi-hop route $fc_{s}-mc_{1}-fc_{d}$ to MC BS $mc_{1}$ and then to the femtocell destination node $fc_{d}$. The traffic intensity $\Psi_{t_{n}}^{k_{n}}\in \Psi =\left\{\Psi_{t_{1}}^{k_{1}},\Psi_{t_{2}}^{k_{2}},\ldots ,\Psi_{t_{\left|N\right|}}^{k_{\left|K\right|}}\right\}$ is one of the parameters that source nodes consider before selecting a route. The CC and the MC BSs are located in the control plane $C^{cc}$, while the FC BSs are located in the data plane $D^{fc}$.

A source node selects a route based on the PU activities, which can either be ON (busy) or OFF (idle). The *ON*/*OFF* duration of PUs in channel $c_{C}\in C$ and link $L=\{l_{1},l_{2},\ldots ,l_{|}n|\}$ follows the *Poisson model*, which is exponentially distributed with rates $\lambda_{ON}^{c_{C},PU}$ for active time and $\lambda_{OFF}^{c_{C},PU}$ for idle time of the *PU* activities, respectively. The channel availability $\Phi_{t,OFF}^{c_{C},l_{n}}$ determines the average availability of the link in the time instant *t* as follows [54]:

(2) $$\begin{array}{cc} \Phi_{t,OFF}^{c_{C},l_{n}}= & \frac{\lambda_{ON}^{c_{C},PU}}{\lambda_{ON}^{c_{C},PU}+\lambda_{OFF}^{c_{C},PU}}+\\ & \frac{\lambda_{OFF}^{c_{C},PU}}{\lambda_{ON}^{c_{C},PU}+\lambda_{OFF}^{c_{C},PU}}\times e^{-{\left(\lambda_{ON}^{c_{C},PU}+\lambda_{OFF}^{c_{C},PU}\right)}^{t}} \end{array}$$

The *PU* and SU avoid any possible collision, but if that happens, it has to be less than the IEEE requirement [45]. In this setup, the appearance of PUs follows the Poisson model that creates a random pattern for ON and OFF. The SU node estimates the average channel available time $\Phi_{t,OFF}^{c_{C},l_{n}}$ of channel $c_{C}\in C$ on a link of a route $k_{n}\in K$. This ON–OFF time assignment is exponentially distributed and shows the duration of the PU transitions (the traffic in each channel). The duration of this random appearance follows the ON–OFF time period shown in Table 2. For example, the PU ON time period can be 50 s and the PU OFF time period can be from 50 to 250 s. This allows PUs to utilize their licensed channels whenever they want during their OFF time. Meanwhile, SUs utilize white spaces opportunistically.

The time horizon in a channel for SU is segregated into the sense–transmit time window [55]. The sensing time is the duration of the channel sensing and processing time. The *processing time* is the duration that the USRP/GNU radio takes for hardware and software initiation, such as packet encoding and decoding, digital conversion, and transmission or reception. The *transmit time* is the duration that a SU node takes to send or receive a data packet.

### 3.1. Reinforcement with Static Learning

In traditional RL (refer to Equation (3)), the learning mechanism has a constant rate that can be determined based on the importance of the current or discount values. The SU receives its reward based on the channel availability (or white spaces, or the idle status of PU). Specifically, the learning rate can be higher (lower) when the PU activity level is lower (higher), as represented by the equation below [46]:

(3) $$\begin{array}{c} Q_{t+1}^{i}\left(s_{t}^{i},a_{t}^{i}\right)=[\left(1-\alpha \right)\times Q_{t}^{i}\left(s_{t}^{i},a_{t}^{i}\right)+\\ \alpha \times \min(r_{t+1}^{i}\left(s_{t+1}^{i}\right))] \end{array}$$

where $r_{t+1}^{i}\left(s_{t+1}^{i}\right)$ is the traffic intensity, which refers to the channel available time of a route $k_{n}\in K$. The traditional RL approach can be ideal for a less dynamic network with low PU activity levels so that no adjustment is required for learning.

### 3.2. Enhanced Reinforcement with Dynamic Learning

For a more dynamic network with frequent changes in the PU appearance in channels, an enhanced RL approach is required to adapt its learning to the dynamicity of the channel. For instance, the dynamicity of MC is less than that of the FC. Therefore, from MC to FC, the dynamicity increases, and the learning rate α is decreased. Equation (3) is enhanced as follows [46]:

(4) $$\begin{array}{c} Q_{t+1}^{i}\left(s_{t}^{i},a_{t}^{i}\right)=\left(1-\alpha \left(s_{t}^{i},a_{t}^{i}\right)\right)\times Q_{t}^{i}\left(s_{t}^{i},a_{t}^{i}\right)+\\ \alpha \times \min(r_{t+1}^{i}\left(s_{t+1}^{i}\right))] \end{array}$$

As the bottleneck link in a route has the least channel capacity, it helps in determining the priority of the D2D route over other routers in the network. A link with a lower channel capacity has a lower priority compared to other routes. The dynamic learning rate is $\alpha_{\min}\leq \alpha \left(s_{t}^{i},a_{t}^{i}\right)\leq \alpha_{\max}$, and it is determined by the availability of channels (or white spaces) as follows:

(5) $$\alpha_{t}^{i}\left(s_{t}^{i},a_{t}^{i}\right)=\Phi_{t,OFF}^{c_{n},l_{i_{n},j_{n}}}$$

For $\alpha_{t}^{i}\left(s_{t}^{i},a_{t}^{i}\right)$, a higher value shows a higher dependency of the Q-value on the current knowledge and a lower value shows a higher dependency of the Q-value on previous knowledge. The dynamic learning of $\alpha_{t}^{i}\left(s_{t}^{i},a_{t}^{i}\right)$ can be varied based on the immediate state–action pair and previously learned rewards. In this study, the agent is myopic and relies on the immediate state–action pair with some consideration of the previously learned Q-value (rather than the next state–action pair (or discounted reward) due to the random appearance of PUs; hence, the discount value is γ=0. Due to the need for interaction with the operating environment, the majority of the testbeds using USRP [33,37,46] have adopted Q-learning to adapt to the environment. Q-learning is the preferred approach because: (a) it does not need datasets required in supervised machine learning approaches [24,25,26] and (b) it provides definite outcomes, particularly whether a channel is utilized or unutilized, which is preferred compared to unsupervised machine learning. The non-learning approach, called non-RL, selects routes based on their priority (e.g., based on the number of hops of the routes specifically routes $k_{2}>k_{3}>k_{4}$). Hence, the proposed dynamic Q-learning approach is compared with the traditional Q-learning approach and the non-learning approach.

In the following sections, the learning model and algorithm of CenTri are presented.

## 4. CenTri: Reinforcement Learning Model and Algorithm

In CenTri, the BSs of both macrocell and small-cell layers collaborate to perform the route selection. A *proactive* link selection mechanism is deployed in the control plane and a *reactive* route selection mechanism is deployed in the data plane.

**Figure 3:**
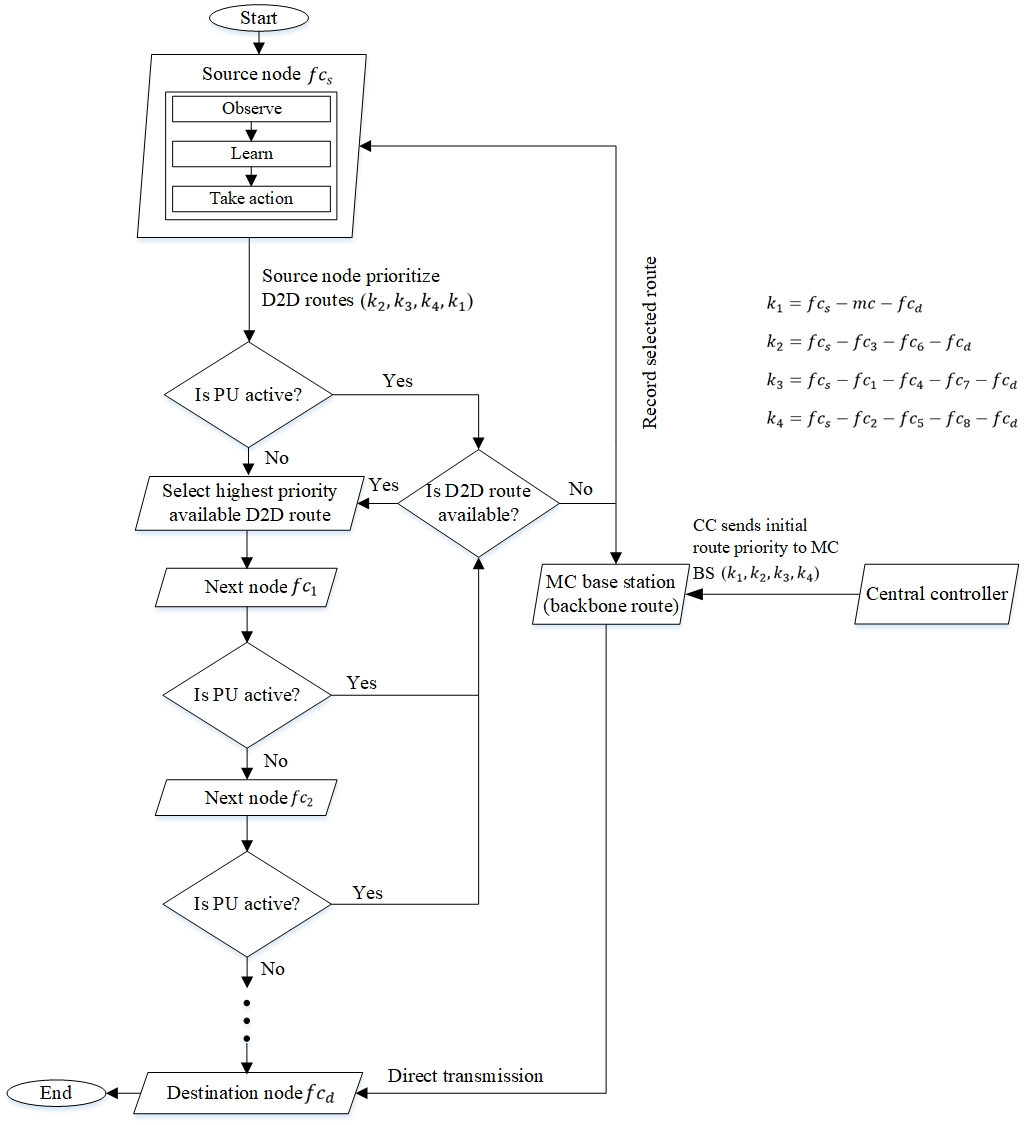
Route selection decision made by the source node based on RL.

Figure 3 presents the route selection mechanism of the RL model, which serves as the decision-making engine. The CC in the control plane selects routes based on factors with less dynamicity (i.e., a lower number of intermediate nodes and delay); while the nodes in the data plane (i.e., the source node $fc_{s}$) select routes based on the priority levels of D2D communication (or with less PU interference). The amount of the delay is higher in D2D routes, so the *distributed RL model* embedded in the BSs and nodes of small cells aims to improve the routing decision made by the CC and select routes with lower PU activity levels and the number of intermediate nodes.

The CC establishes backbone routes in order to provide an always available route for small-cell nodes. The backbone routes are shared among BSs in the data plane and, subsequently, the BSs share the backbone routes with source nodes. The distributed RL model determines the traffic intensity of available routes and establishes routes with the least traffic congestion via traffic offloading. BSs in small cells are part of the backbone routes from the macrocell, and so they inform the CC about their usage of white spaces. Routes are assumed to be disjointed in this paper for simplicity. The rest of this section presents the RL models and algorithms for CenTri.

### 4.1. Reinforcement Learning Models

This section presents the centralized proactive route selection mechanism with the distributed reactive RL model. While the CC establishes more stable routes and backbones, the distributed RL model learns to offload traffic from MC BSs in a collaborative manner.

#### 4.1.1. Centralized Route Selection

In the centralized mechanism, a proactive route selection mechanism is deployed in the CC to establish backbone routes among MC BS and femtocell nodes fc. Backbone routes, which do not have PU activities, help to maximize the packet delivery ratio in networks. The CC evaluates the available routes based on network-wide information gathered from the BSs and femtocell nodes fc. The routes are prioritized as $k_{1}=fc_{s}-mc-fc_{d}$, $k_{2}=fc_{s}-fc_{3}-fc_{6}-fc_{d}$, $k_{3}=fc_{s}-fc_{1}-fc_{4}-fc_{7}-fc_{d}$, and $k_{4}=fc_{s}-fc_{2}-fc_{5}-fc_{8}-fc_{d}$. The evaluated routes from the CC are prioritized based on the number of hops without taking PU activities into consideration. The CC tends to give priority to the shortest route (i.e., the backbone route), which goes through MC BS. The routes are given to source nodes proactively to save processing times incurred in determining routes. Figure 3 shows that the proactive routes are provided by the CC to distributed source nodes in the data plane.

The source node re-prioritizes (or reranks) the given routes based on D2D communication with the minimum number of intermediate nodes (or the number of hops) and traffic intensity. Hence, the source node gives the lowest priority to the backbone route since it does not use D2D communication; this helps in distributing traffic from the MC BS. The presence of PUs has a direct impact on the throughput and delays the performance of packet transmission. Routes with lesser channel switches have lower signaling overheads, leading to higher stability and bandwidth availability [56].

#### 4.1.2. Distributed Reinforcement Learning Model

In the data plane, the distributed RL model is embedded in all small-cell BSs and their corresponding nodes in the network. Hence, a source node in the network can select a route towards the destination in a reactive manner. Route selection in the data plane of the small cells follows the priority levels (refer to Section 1.3). The selected route has lower intermediate nodes and traffic intensity for maximizing traffic offload and achieving a higher throughput through increasing packet delivery rate. The channel capacity of a link in a route is determined by $\Phi_{t_{n}}^{c_{n},k_{n}}$ from Equation (2) and it shows the utilization of links $l_{n}$, including PU activities, in a D2D route $k_{n}$. Assume that a packet $p_{t}^{\varsigma^{i}}\in PT$ with size $\varsigma_{i}$ traverses along a link $l_{n}$ with channel $C_{n}$ at time instant $t_{n}$, the utilization $U_{t_{n}}^{l_{n}k_{n}}$ of links $l_{n}$ of route $k_{n}$ is defined as follows [57]:

(6) $$U_{t_{n}}^{c_{n}l_{n}k_{n}}=\frac{\Sigma p_{t}^{\varsigma^{i}}c_{n}l_{n}}{BW_{c_{n}\in C}}$$

where $BW_{c_{n}}$ is the available bandwidth of the channel $c_{n}$ for the link $l_{n}$ in the femtocell layer. It is noteworthy that all D2D routes in this study have the same bandwidth but different frequencies, which means the link between two nodes uses different channels. The channel utilization of a route is defined as follows:

(7) $$\tau_{t_{n}}^{c_{n}k_{n}}=\Sigma \left(U_{t_{n}}^{c_{n}k_{n}}\right)$$

where $\Sigma (U_{t_{n}}^{c_{n}k_{n}})$ is the sum of the channel utilization by PUs in the links $l_{n}$ of route $k_{n}$ with channels $c_{n}$ at time instant $t_{n}$. The channel utilization of a route includes the PU activities, and it is important for source nodes in the network to be aware of the traffic intensity of each route to reduce the possibility of interference from SUs to PUs.

Traffic intensity is particularly important when multiple routes from a source node have the same number of hops (or intermediate nodes). In the D2D type of communication, including node-to-node, node-to-BS, and BS-to-BS communications, the traffic intensity of a route can be calculated as long as the backbone routes are not utilized. For instance, in Figure 2, the source node $fc_{s}$ and the destination node $fc_{d}$ are three hops away in route $k_{2}$ ($fc_{s}-fc_{3}-fc_{6}-fc_{d}$). However, for routes $k_{3}$ ($fc_{s}-fc_{1}-fc_{4}-fc_{7}-fc_{d}$) and $k_{4}$ ($fc_{s}-fc_{2}-fc_{5}-fc_{8}-fc_{d}$), both routes have the same number of intermediate nodes and links (i.e., four hops). Hence, traffic intensity is used to select a route with a lower congestion level. In this example, PUs appear in route $k_{3}$, then route $k_{4}$ is used. In such a case, route $k_{4}$ continues to be used until communication ends or interference occurs. However, the presence of PUs in a route (i.e., $k_{3}$) makes the route unavailable and recorded as occupied. For a source node that is required to send a data stream to a destination node, it has the initial roadmap comprised of available routes toward the destination node. These routes include nodes and BSs from both macrocell and femtocell layers. Based on the communication priority levels, the source node rearranges the given list by the CC and prefers routes using the femtocell layer. The higher the number of intermediate nodes, the greater the number of links in the route and the more possibility of PUs appearing in its channels. Hence, the route with a lower number of intermediate nodes tends to be selected. A route with a lower PU activity level in the channels of a route has a higher availability, and so the priority level of the route increases, which makes the route more likely to be selected by the source node with an increased learning rate $\alpha (s_{t}^{k_{n}},a_{t}^{k_{n}})$. The given routes with a different number of intermediate nodes, links, and channels experience different PU activity levels. The amount of time that a PU appears in a channel determines the channel capacity at the bottleneck link of a route as follows:

(8) $$\Phi_{t_{n},\beta ,OFF}^{c_{n},k_{n}}=\max_{\forall k_{n}\in K}\left(\min_{l_{n}\in L}\left(\Phi_{t_{n},OFF}^{c_{n},l_{n},k_{n}}\right)\right)$$

where β signifies the bottleneck link of a route. The traffic intensity $\Psi_{t_{n}}^{k_{n}}$ of a route $k_{n}$ in the femtocell layer at time instant $t_{n}$ is the cumulative of the bottleneck channel capacity in the absence of PUs, as follows:

(9) $$\Psi_{t_{n}}^{k_{n}}\leftarrow \Sigma \left(\Phi_{t_{n},\beta ,OFF}^{c_{n},k_{n}}\right)$$

The traffic intensity of a route is proportional to its channel capacity. A greater channel capacity in a route has lesser PU interference and vice-versa. The source node learns about the dynamic changes of traffic intensity and adapts the learning rate accordingly. For instance, the greater traffic intensity in route $k_{1}$ at the time instant $t_{1}$ causes the source node to reduce the learning rate $\alpha (s_{t_{1}}^{k_{1}},a_{t_{1}}^{k_{1}})\to 0$ closer to zero, which makes the Q-function more dependent on the previous Q-value. The lower traffic intensity causes the source node to increase the learning rate $\alpha (s_{t_{1}}^{k_{1}},a_{t_{1}}^{k_{1}})\to 1$ closer to one, which makes the Q-function more dependent on the current Q-value. The source node makes a route selection decision by choosing a route with the maximum Q-value, which has the minimum PU activities as follows:

(10) $$a_{t}^{k_{n}}=\max_{k_{n}\in A}\left(Q_{t_{n}}^{k_{n}}\left(s_{t_{n}}^{k_{n}},k_{n}^{fc}\right)\right)$$

The dynamic learning rate $\alpha_{\min}\leq \alpha \left(s_{t_{n}}^{k_{n}},a_{t_{n}}^{k_{n}}\right)\leq \alpha_{\max}$ is as follows:

(11) $$\alpha \left(s_{t_{n}}^{k_{n}},a_{t_{n}}^{k_{n}}\right)\leftarrow \Psi_{t_{n}}^{k_{n}}$$

A source node is equipped with the Q-routing model to rank the D2D routes. The best route toward the destination node is based on the number of intermediate hops and traffic intensity of available routes. Table 1 shows the RL model of the reactive mechanism embedded in BSs and nodes. In this model, state $s_{t}^{k_{n}}$ represents the given routes $k_{n}\in K$ from the CC towards the destination node $fc_{d}$, in which both the source and destination nodes are in the data plane of the femtocell. Action $a_{t}^{k_{n}}$ represents the selection of an available route with the highest priority. If one of the selected routes is blocked by PUs, then the next highest priority route is selected by the source node. Reward $r_{t}^{k_{n},t+1}$ represents the cost reflecting the traffic intensity of a route from the source node to the destination node.

Based on Table 1, three criteria are checked between source and destination nodes prior to communication in the data plane: (a) the type of communication (i.e., D2D); (b) the number of intermediate nodes; and (c) traffic intensity. The first criterion uses the available white spaces in channels and intends to offload traffic from MC BS. The second criterion helps to make route decisions more efficient by looking at routes with a lesser number of intermediate nodes, which helps to reduce the possibility of the PU appearance, leading to a higher successful transmission rate. The third criterion selects the route with fewer PU activities when two routes are identical in terms of the number of hops and channels. The route with fewer PU activities has a lower traffic intensity and a higher Q-value, so it is preferred.

**Figure 4:**
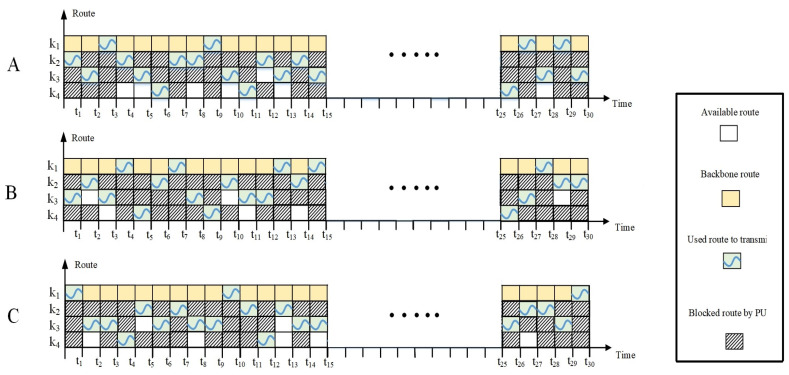
Random appearance of PU activities in D2D routes in three transmission cycles (**A**–**C**).

In Figure 4, an example of the random appearance of PUs in routes is illustrated for three transmission cycles, namely A, B, C. The PUs can occupy a channel of routes $k_{2}$, $k_{3}$, and/ or $k_{4}$. The presence of PUs in one of the link channels of a D2D route can cause the entire route to be blocked, and the source node must select another route. In this figure, at time *t*, the source node selects a route to the destination node. Since routes $k_{3}$ and $k_{4}$ are occupied by PUs, route $k_{2}$ is selected. At time $t_{1}$, both routes $k_{2}$ and $k_{4}$ are occupied; therefore, route $k_{3}$ is selected. At time $t_{2}$, all D2D routes $k_{2}$, $k_{3}$, and $k_{4}$ are occupied, so the source node uses the backbone route through MC BS. At time $t_{3}$, route $k_{3}$ is occupied by PUs, but both routes $k_{2}$ and $k_{4}$ are available to the source node. The source node selects route $k_{2}$ as it has a higher priority due to a smaller number of intermediate hops and nodes compared to route $k_{4}$. At time $t_{4}$, route $k_{2}$ is occupied by PUs, and both routes $k_{3}$ and $k_{4}$ are available to the source node. In this case, the source node selects route $k_{3}$ as it has a higher priority over route $k_{4}$. During the communication between the source node and the destination node through D2D routes, the source node learns about the appearance of PUs and the availability of D2D routes. This makes route selection more accurate as time goes by. For instance, both D2D routes $k_{3}$ and $k_{4}$ have the same number of hops, but the source node has learned that route $k_{3}$ has a higher channel capacity, and so it has a higher Q-value compared to route $k_{4}$. Therefore, route $k_{3}$ is selected.

### 4.2. Reinforcement Learning Algorithm

**Figure 5:**
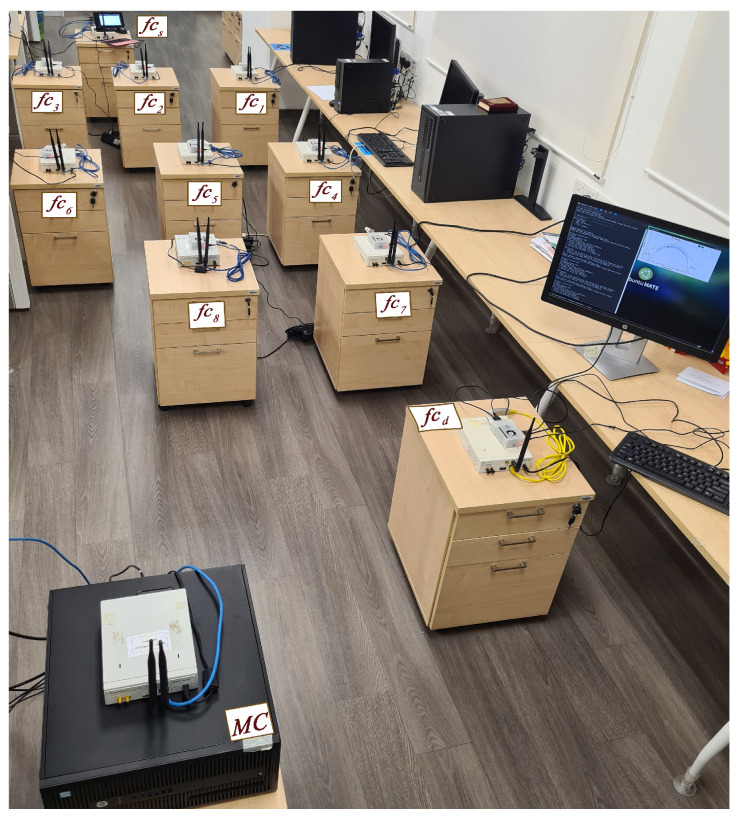
Experimental setup for the hybrid route selection with three D2D routes and a backbone route via MC BS.

In this section, the RL algorithm for the distributed mechanism is presented. All the nodes and BSs of small cells in the data plane are equipped with the RL algorithm and receive a route map from the CC proactively. This enables them to select the best route proactively based on priority levels (see Section 4.1.2), which helps to offload traffic from MC BS. In the data plane of the small cell, a source node transmits a data stream to a destination node in a selected route out of the given routes by the CC. During the transmission, if the route is interrupted by PUs, a second prioritized route is selected and the previous route is identified as a route with a high traffic intensity level. By continuing this process, a table is updated with route scores based on channel capacity at the end of each transmission cycle, which gives a clearer pattern of the random appearance of PUs. An experimental setup and configuration are shown in Figure 5. In this platform, since the network layer is the focus of this study, the physical distance between nodes, as well as phenomena, such as shadowing and fading, are not the concerns in this work.

Algorithm 1 provides a general route selection scheme in which the CC sends the initial prioritized routes through the MC BS to the source node in the data plane proactively. As for CC, it is assumed that routes are readily available and prioritized based on the number of hops.
**Algorithm 1** General description of the route selection by the source node.1:**procedure**hybrid route selection2:     CC sends initial prioritized routes to MC BS proactively3:     MC BS sends prioritized routes *K*, where $k_{1}$ has the highest and $k_{k}$ has the lowest priority, to a source node $fc_{s}$4:    **for** ($k_{D2D}\in K$) **do**5:         Select route $k_{1}$ if it has the least hops and traffic intensity Ψ at time instant *t* when PU is OFF6:        **if** all $k_{D2D}$ have ON PUs **then**7:            Select the backbone route $k_{bb}$8:        **end if**9:    **end for**10:**end procedure**

Algorithm 2 shows the distributed route selection mechanism for traffic offloading. Based on the flowchart shown in Figure 3, the source node receives a route map (i.e., a list of routes created proactively), which is prioritized based on the number of hops, from the CC. The source node rearranges the list and gives priority to D2D routes in order to offload traffic from MC BS. The Q-value is dependent on traffic intensity, which is based on the channel capacity of the links of a route and the learning rate $\alpha_{t}^{i}\left(s_{t}^{i},a_{t}^{i}\right)$ that changes dynamically based on the PU activity level. Therefore, routes with lesser PU activities tend to be selected compared to those with higher PU activities.
**Algorithm 2** RL mechanism at distributed nodes and BSs.1:**procedure** D2D Route selection2:     /* Source node selects a route to offload traffic from MC BS */3:     MCBS receives a route map (i.e., $\left\{k_{bb},k_{2},k_{3},k_{4}\right\}$ from the CC and sends it to $fc_{s}$4:     /* Stage 1 */5:    **for** time $t_{1}$; $fc_{s}$ reprioritize D2D routes in the route map **do**6:        $k=\{k_{2}>k_{3}>k_{4}>k_{bb}\}$7:        **if** $k_{2}$ or $k_{3}$ or $k_{4}$ is not available **then**8:            $fc_{s}$ use backbone route $k_{bb}$9:        **end if**10:    **end for**11:     /* Stage 2 */12:    **for** time $t_{n+1}$, n∈|N|; $fc_{s}$ reprioritize D2D routes based on traffic intensity $\Psi_{t_{n}}^{k_{n}}$ and Equation (4) **do**13:        Estimate $\Phi_{t_{n},OFF}^{c_{n},l_{n},k_{n}}$ using Equations (2) and (8)14:        **if** $\Psi_{t_{n}}^{k_{n}}<\Phi_{t_{n},\beta ,OFF}^{c_{n},k_{n}}$ /* traffic intensity is not updated */ **then**15:           update $\Psi_{t_{n}}^{k_{n}}\leftarrow \Sigma \left(\Phi_{t_{n},\beta ,OFF}^{c_{n},k_{n}}\right)$16:        **end if**17:        Calculate dynamic learning rate $\alpha (s_{t_{n}}^{k_{n}},a_{t_{n}}^{k_{n}})$ using Equation (10)18:        Update the Q-value using Equation (4)19:        **for** $fc_{s}$, **do** select a D2D route with the maximum Q-value from routes $\left\{k_{2}>k_{3}>k_{4}\right\}$20:        **end for**21:    **end for**22:**end procedure**

#### 4.2.1. Implementation Requirements and Parameters

The implementation has eleven USRP/GNU radio units as nodes and BSs. Each of the ten USRP/GNU radio units is connected with a Raspberry Pi3 B+ unit equipped with 30 GB of external memory for storing and running algorithms. The USRP unit, specifically USRP N200, is equipped with the VERT900 antenna, and the GNU radio runs an open-source software-defined radio (SDR). A personal computer, which is equipped with the core i7 processor and 16 GB RAM, serves as MC BS. The D2D nodes have closer proximity among themselves compared to MC BS, so the transmission power is 10 dBm (10 mW) among themselves and 20 dBm (100 mW) with the MC BS.

Table 2 presents the parameters. In this platform, the user datagram protocol (UDP) is the preferred transport layer protocol for multimedia applications because it is connectionless and it does not perform retransmission during packet loss, which reduces delay at the expense of the acceptable packet loss. Figure 5 shows the platform with USRPs equipped with RP3. Nodes are located in the MC BS proximity and receive route information proactively from the CC via MC BS.

#### 4.2.2. Assumptions

The platform performs multi-hop communication from the source node to the destination node. There are a few assumptions in this setup as follows:

- The delay incurred in multi-hop communication is not considered in order to focus on routes with less traffic (i.e., with low PU activities).
- The backbone and D2D routes are readily available, and the source node re-prioritizes them.
- A D2D route is up to three hops, and the source and destination nodes do not have direct communication.

#### 4.2.3. Appearance of PUs on Channels

Three PUs reappear in the operating channels of the D2D communication randomly. The backbone route $k_{1}$, which serves as a backup, is free from PU activities. When the channel of a route has PU activities, the route breaks and the source node must select another available route following the priority mechanism explained in Section 4.1.1. There are three scenarios related to the presence of PU activities in routes $k_{2}$, $k_{3}$, and $k_{4}$. In all scenarios, the destination node is beyond the transmission range of the source node, and so the traffic stream must go through the intermediate nodes of the network.

##### Scenario 1

**Figure 6:**
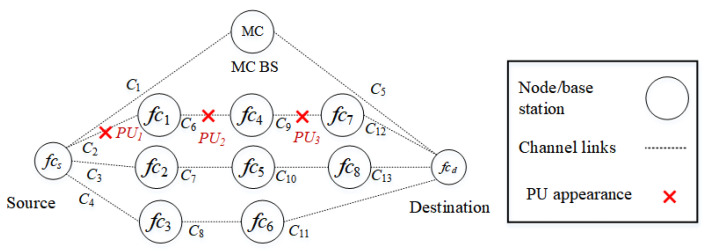
Scenario 1: The appearance of PUs in route $k_{3}$ interfere with channels $c_{2}$, $c_{6}$, and $c_{9}$.

In the first scenario, as shown in Figure 6, PUs reappear in route $k_{3}$ ($fc_{s}-fc_{1}-fc_{4}-fc_{7}-fc_{d}$) in a random manner. $PU_{1}$, $PU_{2}$, and $PU_{3}$ interfere with channels $c_{2}$, $c_{6}$, and $c_{9}$, respectively. The source node selects either D2D routes $k_{2}$ or $k_{4}$, or the backbone route $k_{1}$. Since route $k_{2}$ has a higher priority due to a lower number of hops, it is selected.

##### Scenario 2

**Figure 7:**
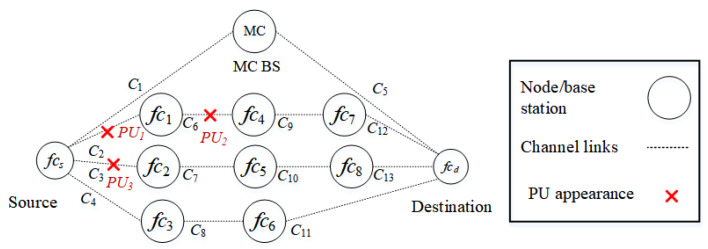
Scenario 2: The appearance of PUs in routes $k_{3}$ and $k_{4}$ interfere with channels $c_{2}$, $c_{3}$, and $c_{6}$.

In the second scenario, as shown in Figure 7, PUs reappear in routes $k_{3}$ ($fc_{s}-fc_{1}-fc_{4}-fc_{7}-fc_{d}$) and $k_{4}$ ($fc_{s}-fc_{2}-fc_{5}-fc_{8}-fc_{d}$). $PU_{1}$, $PU_{2}$, and $PU_{3}$ interfere with channels $c_{2}$, $c_{6}$, and $c_{3}$, respectively. Specifically, two channels, $c_{2}$ and $c_{6}$ of route $k_{3}$ and channel $c_{3}$ of route $k_{4}$, are occupied by PUs. The source node selects the D2D route $k_{2}$ rather than the backbone route $k_{1}$.

##### Scenario 3

**Figure 8:**
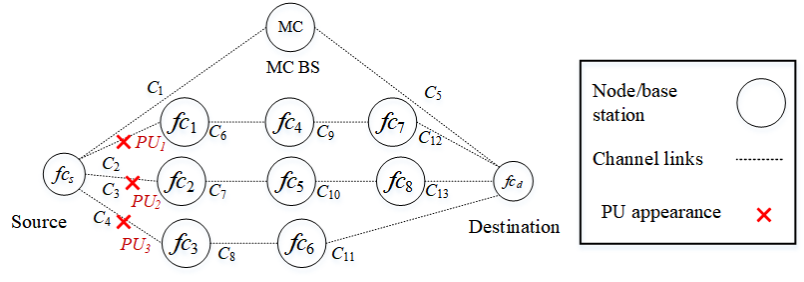
Scenario 3: The appearance of PUs in routes $k_{2}$, $k_{3}$, and $k_{4}$ interfere with channels $c_{2}$, $c_{3}$, and $c_{4}$.

In the third scenario, as shown in Figure 8, PUs reappear in all D2D routes, including routes $k_{2}$ ($fc_{s}-fc_{3}-fc_{6}-fc_{d}$), $k_{3}$ ($fc_{s}-fc_{1}-fc_{4}-fc_{7}-fc_{d}$), and $k_{4}$ ($fc_{s}-fc_{2}-fc_{5}-fc_{8}-fc_{d}$). $PU_{1}$, $PU_{2}$, and $PU_{3}$ interfere with channels $c_{2}$, $c_{3}$, and $c_{4}$, respectively. Specifically, channel $c_{2}$ of route $k_{3}$, channel $c_{3}$ of route $k_{4}$, and channel $c_{4}$ of route $k_{2}$ are occupied by PUs. Only the backbone route $k_{1}$ is available to the source node.

## 5. Results and Discussion

Simulation results, including the packet delivery ratio, end-to-end delay, throughput, and the number of route breakages, are presented.

### 5.1. Packet Delivery Ratio

**Figure 9:**
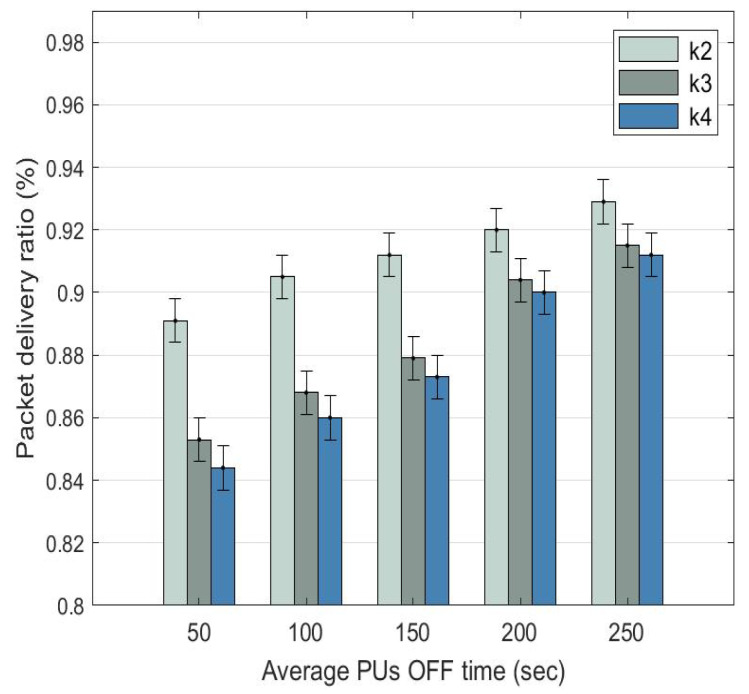
Average packet delivery ratio comparison among D2D routes with dynamic α at different PU OFF time.

The packet delivery ratio (PDR) is the ratio of the number of packets received by the destination node to the number of packets sent by the source node. In Figure 9, PDR increases for D2D routes $k_{1}$, $k_{2}$, and $k_{3}$ as the PU OFF time increases. When the PU OFF time increases from 50 to 250 s, the PDR of: (a) route $k_{2}$ increases from 0.891 (89.1%) to 0.929 (92.2%); (b) route $k_{3}$ increases from 0.853 (85.3%) to 0.915 (91.5%); and (c) route $k_{4}$ increases from the lowest at 0.844 (84.4%) to 0.912 (91.2%).

Route $k_{2}$ achieves a better PDR compared to routes $k_{3}$ and $k_{4}$ since it has a lower number of hops and PU activities. For routes $k_{3}$ and $k_{4}$, their PDRs are very close to each other and their gap reduces as the PU OFF time increases. This is because both routes have the same number of hops; however, route $k_{3}$ has a lesser presence of PUs, explaining why it is a preferred route over route $k_{4}$.

### 5.2. End-to-End Delay

The end-to-end delay is the time taken by a data stream to be transmitted from the source node $fc_{s}$ to the destination node $fc_{d}$. The three D2D routes have different numbers of intermediate nodes (or hops). Route $k_{2}=fc_{s}-fc_{3}-fc_{6}-fc_{d}$ has four nodes with two intermediate nodes and routes $k_{3}=fc_{s}-fc_{1}-fc_{4}-fc_{7}-fc_{d}$ and $k_{4}=fc_{s}-fc_{2}-fc_{5}-fc_{8}-fc_{d}$ have five nodes with three intermediate nodes. Route $k_{2}$ has the lowest end-to-end delay among the D2D routes since it has the lowest number of intermediate nodes. Since routes $k_{3}$ and $k_{4}$ have the same number of intermediate nodes, the source node selects one over the other through the learning mechanism, explained in Section 4.2.3.

**Figure 10:**
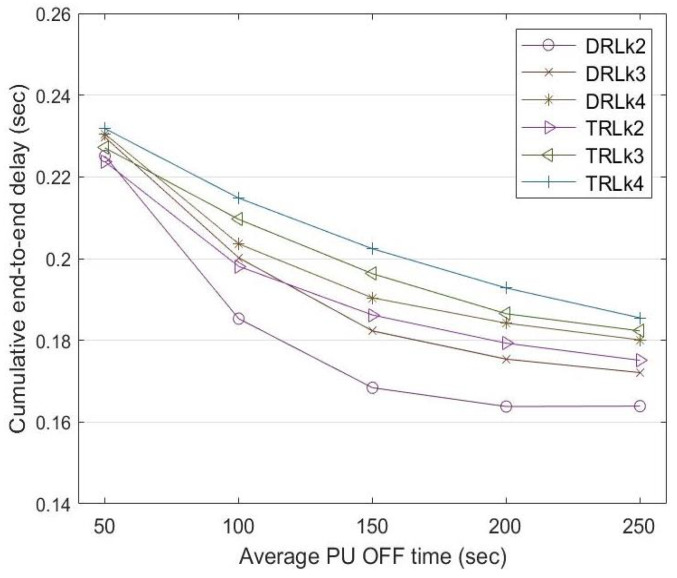
End-to-end delay comparison among routes $k_{2}$, $k_{3}$, and $k_{4}$ between DRL and TRL with α=0.5 at a different PU OFF time.

Figure 10 shows a comparison of the end-to-end delay incurred between the traditional reinforcement learning (TRL) mechanism with α=0.5 and the dynamic reinforcement learning (DRL) mechanism. In the traditional RL mechanism, the learning rate is constant at α=0.5 for the entire experiment. The end-to-end delay reduces with increasing PU OFF time. Compared to traditional reinforcement learning, dynamic reinforcement learning shows a lower end-to-end delay for routes $k_{2}$, $k_{3}$, and $k_{4}$ when the PU OFF time increases from 50 to 250 s.

### 5.3. Throughput

**Figure 11:**
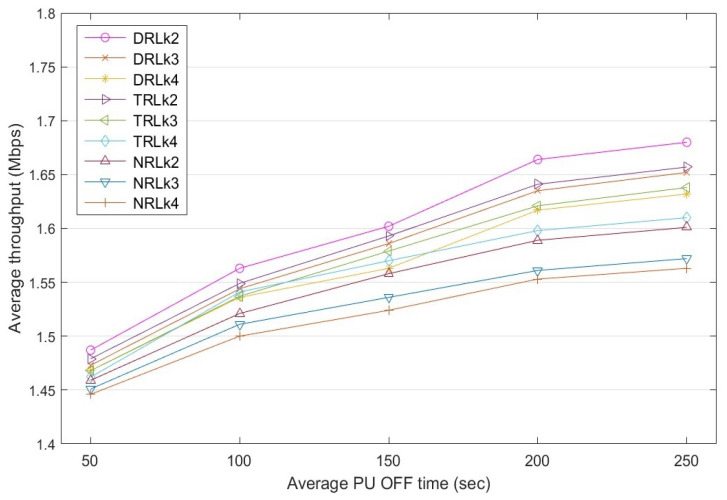
Average throughput comparison among routes $k_{2}$, $k_{3}$, and $k_{4}$ for dynamic RL with dynamic α, traditional RL with α=0.5 and non-RL at a different PU OFF time.

Throughput is the rate of the successful data stream delivered to the destination node through a selected route in a specific time frame. Figure 11 shows a comparison of the throughput achieved by the three D2D routes $k_{2}$, $k_{3}$, and $k_{4}$ for different PU OFF times. Based on the experimental results, the average throughput of the routes increases when the PU appearance reduces. Therefore, increasing the PU OFF time has a positive effect on the rate of the data stream transmission. The throughput of the dynamic reinforcement learning (DRL) mechanism, which has a dynamic learning rate α that varies with rewards, is affected by the PU appearance in a route. Specifically, when the PU OFF time increases from 50 to 250 s: (a) the throughput of route $k_{2}$ increases from 1.487 to 1.68 Mbps, (b) the throughput of route $k_{3}$ increases from 1.473 to 1.652 Mbps, and (c) the throughput of route $k_{4}$ increases from 1.468 to 1.642 Mbps. A similar trend is observed in: (a) traditional reinforcement learning (TRL) with a fixed learning rate of α=0.5; and (b) the non-learning approach, called non-RL (NRL), which selects routes using their priority $k_{2}>k_{3}>k_{4}$, which is based on the number of hops of the routes. Overall, DRL outperforms both TRL and NRL.

### 5.4. Number of Route Breakages

The source node selects a route based on the priority given by the CC. However, the priority of routes with D2D communication changes with the presence of PUs and successful data transmission to the destination node. For each data transmission cycle, a route breakage occurs when a PU reappears in a selected route, and this causes the source node to switch to another route.

**Figure 12:**
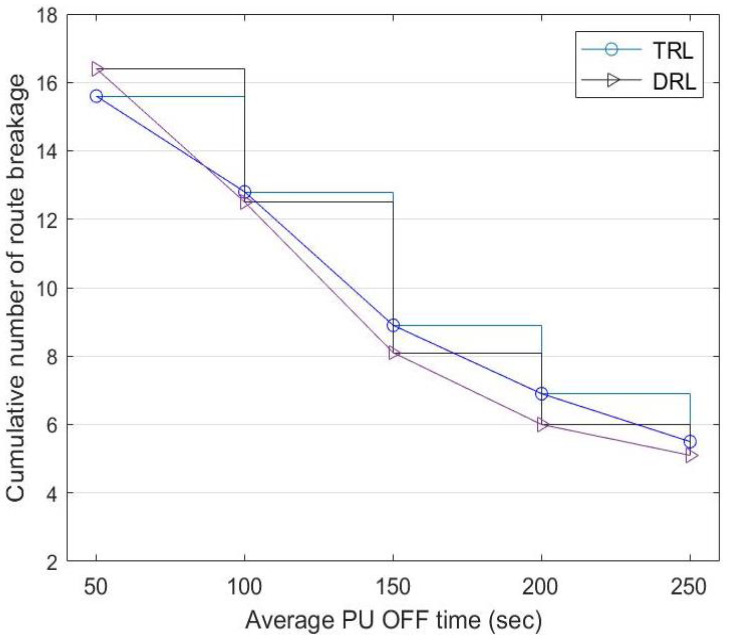
Cumulative number of route breakages between TRL with α=0.5 and the DRL mechanism at a different PU-OFF time.

Figure 12 shows a comparison of the cumulative route breakage between DRL and TRL with a fixed α=0.5. Although TRL has a better performance with less route breakage at the beginning, the source node learns more about routes with higher successful transmission rates as time goes by, contributing to the improvement in DRL. When the PU OFF time increases from 50 to 250 s: (a) the route breakage of TRL reduces from 15.6 to 5.5 and (b) the route breakage of DRL reduces from 16.4 to 5.1.

## 6. Conclusions and Future Work

This paper proposes CenTri, which is a hybrid route selection scheme that uses white spaces to offload traffic from macrocell to small-cell base stations with heterogeneous nodes in 5G network scenarios. It caters to important characteristics of 5G network scenarios, including the dynamicity of channel availability, heterogeneity, and ultra-densification. In this paper, device-to-device (D2D) communication uses traditional reinforcement learning (TRL) and dynamic reinforcement learning (DRL) approaches. While TRL uses a constant learning rate, DRL uses a dynamic learning rate that changes with primary user (PU) activity levels. Our work was tested in a testbed with eleven USRP/GNU radio units. Each USRP unit was embedded with a mini-computer called RP3 to provide more realistic scenarios. Compared to TRL, experimental results show improvement in different quality of service (QoS) metrics, including a higher packet delivery ratio, throughput, lower end-to-end delay, and the number of route breakages. Routes with higher intermediate nodes also achieved higher end-to-end delay but a lower packet delivery ratio and throughput.

In the future, CenTri will require more testing with a higher number of routes and intermediate nodes. Better processing units can relax the assumptions made, including the processing delay. Moreover, a cross-layer design for studying the physical data link and network layers will help to provide a more realistic testing environment.

## Figures and Tables

**Table 1:** The RL model for the route selection embedded in the BS of the small cell and its corresponding nodes in the data plane.

State	$s_{t}^{k_{n}}\in S=\left\{k_{1}^{MC},k_{2}^{fc_{1}},k_{3}^{fc_{2}},...,k_{\left|K\right|}^{MC,fc_{n}}\right\}$ represents the given route from the CC at the time instant $t_{1}$.
Action	$a_{t}^{k_{n}}\in A=\left\{k_{t_{1}}^{fc},k_{t_{2}}^{fc},...,k_{t_{n}}^{fc}\right\}$ represents a set of actions in which a source node $fc_{s}$ selects a D2D route $k_{n}\in K$ with the highest priority level at the time instant $t_{1}$.
Reward	$r_{t+1}^{k_{n}}\left(s_{t}^{k_{n}},a_{t}^{k_{n}}\right)$ represents the traffic intensity $\Psi_{n}^{k_{n}}$ of the selected route when PU is in the OFF state at the time instant t+1.

**Table 2:** Experimental setup parameters.

Category	Parameter	Value
Experiment	Duration	900 s
	Number of channels	11
	Number of USRP/GNU radio nodes	10
USRP/GNU radio nodes	Transport layer	UDP
USRP	Channel bandwidth	40 MHz
	Sample rate	1.1 MB
USRP antenna	D2D transmission power among nodes	10 dBm
	Transmission power between node and MC BS	20 dBm
	Carrier frequency	850 MHz
RP3	Operating system	Ubuntu-Mate
PU activities	PU ON time	50 s
	PU OFF time	{50,100,150,200,250}s

## Data Availability

Data sharing not applicable.
